# Impact of dispersion media and carrier type on spray-dried proliposome powder formulations loaded with beclomethasone dipropionate for their pulmonary drug delivery via a next generation impactor

**DOI:** 10.1371/journal.pone.0281860

**Published:** 2023-03-13

**Authors:** Iftikhar Khan, Ali Al-Hasani, Mohsin H. Khan, Aamir N. Khan, Fakhr-e -Alam, Sajid K. Sadozai, Abdelbary Elhissi, Jehanzeb Khan, Sakib Yousaf

**Affiliations:** 1 School of Pharmacy and Biomolecular Sciences, Liverpool John Moores University, Liverpool, United Kingdom; 2 Surgical A Ward, Khyber Teaching Hospital, Peshawar, Pakistan; 3 Cardiology Department, Lady Reading Hospital, Peshawar, Pakistan; 4 Department of Hepatology, King’s College Hospital, Denmark Hill, London, United Kingdom; 5 Department of Pharmacy, Kohat University of Science and Technology, Kohat, Pakistan; 6 Pharmaceutical Sciences Section, College of Pharmacy, Qatar University, Doha, Qatar; 7 Zia Medical Centre, Abu Dhabi, UAE; St. John’s University, UNITED STATES

## Abstract

Drug delivery *via* aerosolization for localized and systemic effect is a non-invasive approach to achieving pulmonary targeting. The aim of this study was to prepare spray-dried proliposome (SDP) powder formulations to produce carrier particles for superior aerosolization performance, assessed *via* a next generation impactor (NGI) in combination with a dry powder inhaler. SDP powder formulations (F1-F10) were prepared using a spray dryer, employing five different types of lactose carriers (Lactose monohydrate (LMH), lactose microfine (LMF), lactose 003, lactose 220 and lactose 300) and two different dispersion media. The first dispersion medium was comprised of water and ethanol (50:50% v/v ratio), and the second dispersion medium comprised wholly of ethanol (100%). In the first dispersion medium, the lipid phase (consisting of Soya phosphatidylcholine (SPC as phospholipid) and Beclomethasone dipropionate (BDP; model drug) were dissolved in ethanol and the lactose carrier in water, followed by spray drying. Whereas in second dispersion medium, the lipid phase and lactose carrier were dispersed in ethanol only, post spray drying. SDP powder formulations (F1-F5) possessed significantly smaller particles (2.89 ± 1.24–4.48 ± 1.20 μm), when compared to SDP F6-F10 formulations (10.63 ± 3.71–19.27 ± 4.98 μm), irrespective of lactose carrier type *via* SEM (scanning electron microscopy). Crystallinity of the F6-F10 and amorphicity of F1-F15 formulations were confirmed by XRD (X-ray diffraction). Differences in size and crystallinity were further reflected in production yield, where significantly higher production yield was obtained for F1-F5 (74.87 ± 4.28–87.32 ± 2.42%) then F6-F10 formulations (40.08 ± 5.714–54.98 ± 5.82%), irrespective of carrier type. Negligible differences were noted in terms of entrapment efficiency, when comparing F1-F5 SDP formulations (94.67 ± 8.41–96.35 ± 7.93) to F6-F10 formulations (78.16 ± 9.35–82.95 ± 9.62). Moreover, formulations F1-F5 demonstrated significantly higher fine particle fraction (FPF), fine particle dose (FPD) and respirable fraction (RF) (on average of 30.35%, 890.12 μg and 85.90%) when compared to counterpart SDP powder formulations (F6-F10). This study has demonstrated that when a combination of water and ethanol was employed as dispersion medium (formulations F1-F5), superior formulation properties for pulmonary drug delivery were observed, irrespective of carrier type employed.

## Introduction

Pulmonary delivery is a non-invasive route and has been historically used for inhalation therapy for decades [1, 2]. Recently, interest in this route has increased significantly with the delivery of micro and nano formulations developed to produce a localized effect, desirable due to the large surface area of lungs (100 m^2^) [3, 4]. The pulmonary route additionally offers low enzymatic activity, a potentially low degradation rate and relatively high absorption rate [3]. For localized effect in the pulmonary system, formulations can be deposited directly onto the lung epithelium, improving rapid onset of action and requiring lower doses.

Nanotechnology and nanocarriers have gained popularity delivering therapeutically active compounds into the lungs, offering a multitude of benefits, including; high drug loading, drug shielding from degradation, controlled drug release and biocompatibility, as well as stability of formulations during the nebulization process. Several lipid-based formulations have successfully delivered therapeutically active ingredients offering the aforementioned benefits into the pulmonary system, examples include; transfersomes and protransfersomes [5–8], niosomes and proniosomes [9, 10], micelles [11], microspheres [12, 13], solid lipid nanoparticles [14] and nanostructured lipid carriers [3, 15].

Lipid-based drug delivery systems are extensively used to deliver hydrophobic drugs. Liposomes are capable of entrapping both hydrophilic (into the central core of vesicles) and hydrophobic drugs (in the concentric bilayers) simultaneously. Liposomes are inherently unstable, this is attributed to the hydrolysis and oxidation of liposomal phospholipids, resulting in vesicles fusion or aggregation and leakage of entrapped drugs. Such instabilities may significantly reduce the shelf-life of liposomal formulations. However, this instability can be overcome by the production of proliposome formulations [16–18]. There are two prominent types of proliposome formulation, these are; particular-based [16, 19] and ethanol-based formulations [18, 20], each formulation generates liposomes *via* the hydration method (i.e., takes place above the phase transition temperature of the phospholipid selected). Particulate-based Proliposome formulations are produced by either a spray-drying method [21], fluidized-bed coating method [22] or a slurry-based method [23]. These formulations in powder proliposome form are more stable and can be hydrated to form liposomes with aqueous media for analysis. Spray-drying is a single step process to convert aqueous dispersions/suspensions into dry powders by controlling several parameters in order to obtained powders with desirable physical characteristics for aerosolization. Conversely, ethanol-based proliposome formulations are essentially concentrated ethanolic solutions containing dissolved phospholipid, which generate liposomes upon hydration/addition of aqueous solvent (i.e., water or buffer) [18]. Such formulations were also employed and deemed suitable for the generation and inhalation of liposomes *via* aerosolization through nebulizers [24, 25]. Proliposomes are therefore stable powder formulations of carbohydrate carrier coated/mixed with phospholipid and drug, which generate liposome vesicles upon simple hydration step.

Moreover, various types of carbohydrate carriers and amino acids can be added to prepare spray-dried formulations with improved aersolization performance [26, 27]. It is vital to select appropriate excipients for inhalation, leading to optimum and functional dry powders, achieving higher deposition in the peripheral airways. On the contrary, larger spray-dried particles or aggregated/fused particles may deposit in the upper respiratory tract, impacting on the diffusion of the vesicles through the epithelial lining of the pulmonary system [28].

Delivery of nanoparticles is an alternative strategy to deliver therapeutic agents into the pulmonary system to treat respiratory disease when compared to the larger microparticles (>5 μm). As, larger microparticles are poorly aerosolized, depositing on the upper respiratory tract, due to their low inertia and poor manoeuvrability in the pulmonary system [29, 30]. Thus, particle size should ideally be within nanometer size range for superior peripheral lung deposition. The incorporation of carbohydrate carriers or excipients and phospholipid may produce particles within in this aerodynamic diameter [6, 15, 31].

Beclomethasone dipropionate (BDP) is a synthetic and an insoluble glucocorticoid steroidal drug with well-established clinical indications. BDP is commercially available on the market, used commonly in the treatment of asthma *via* inhalation (i.e., as prophylaxis) and therefore employed in this study. BDP is available as a dry powder inhaler with a brand name of Asmabec, whereas Clenil and QVAR contain BDP with other excipients as a pressurised metered dose inhaler. A number of different lipid-based dry and aqueous formulations have employed BDP as a model drug for targeting pulmonary system [22]. BDP is a lipophilic drug and entraps in the concentric bilayers of liposome vesicles. However, the entrapment efficiency is based on the method of separation, where the entrapped drug in vesicles bilayers can be separated from the free drug in the suspensions produced; for example using deuterium oxide as a dispersion medium [32, 33], or using Millipore filters with respect to the molecular weight of the drug [6]. The type of phospholipid (i.e., natural or synthetic source) and their hydrocarbon chain length may also affect the entrapment efficiency [6, 9, 34]. Moreover, encapsulation of drug may be affected by the selection of carbohydrate carrier, which may be attributed to the particle’s morphology and their interaction with formulation ingredients [32, 34, 35]. Size reduction *via* probe sonication or extrusion technology processes, as well as the inclusion of excipients in the formulation may alter the entrapment efficiency [5, 7, 36].

For pulmonary drug delivery, many approaches have been adopted/employed. Carbohydrate carriers have been used with drug candidates followed by powder blend, to adhere drug particles on the carbohydrate substrate/carrier [37]. Many other candidates were also employed to deliver drugs into the pulmonary systems. For example, micelles, polymeric and dendrimers [38–40]. However, liposomes as lipid-based formulations have been acknowledged as highly promising delivery carriers and have attained clinical approval [41] and several have reached to the cosmetics market [42, 43]. Moreover, liposome-based products like DaunoXome® and Doxil® were the first formulations to be commercially presented for clinical use [44]. Furthermore, in the arena of inhalation therapy, liposome demonstrated commercialization success where Arikayce® (Insmed, NJ, USA) (amikacin-loaded liposome formulation *via* nebulization) was approved by FDA for the treatment of Mycobacterium avium complex (MAC) lung disease [45].

In this study, various types of proliposome formulations were prepared (*via* spray drying), with aerosolization performance of each formulation conducted *via* a next generation impactor (NGI) for pulmonary drug delivery. The aim of this study was to develop and optimise BDP-loaded spray-dried proliposome (SDP) powder formulations and to investigate the impact of various factors, such as carbohydrate carrier (five different lactose carrier used) and dispersion media (1^st^ dispersion medium consisted of water:ethanol in 50:50% v/v ratio; and the 2^nd^ medium was ethanol alone i.e. 100%) on the physicochemical properties of SDP formulations, including; particle size, particle morphology, production yield, vesicle size, polydispersity index (PDI), Zeta potential and entrapment efficiency. Furthermore, aerosolization performance was performed to determine SDP formulation fine particle dose (FPD), fine particle fraction (FPF) and respirable fraction (RF) to identify the best formulation and dispersion media.

## Materials and methods

### Materials

Beclomethasone dipropionate (BDP) was purchased from Sigma Aldrich, UK. Lactose monohydrate (LMH) was acquired from VWR Chemicals, UK. HPLC-grade methanol and ethanol were acquired from Fisher scientific, UK. Soya phosphatidylcholine (SPC; Lipoid S100; 94% purity) was generously gifted from Lipoid, Germany. Lactose microfine (LMF), lactose 003, lactose 220 and lactose 300 were also gifted from DEF pharma, Germany. Low resistant dry powder inhalers (RS01; 4kPa at 100 L/min) were gifted by Plastiape, Italy. Hydroxypropyl methylcellulose capsule (Size 3) was also gifted by Qualicaps, Spain.

### Spray-dried proliposome formulations using lactose carriers

Spray-dried proliposome (SDP) powders of lactose carriers (lactose monohydrate (LMH), lactose microfine (LMF), lactose 003, lactose 220 and lactose 300) were prepared *via* spray drying (Büchi B-290 Mini Spray Dryer, Switzerland), where an inlet temperature of 120°C, an airflow rate of 601 L/h, pump feed rate of 15% were maintained to prepare 10 formulations (i.e., F1 –F10) (Table 1). BDP was used as the model drug in the SDP formulations.

**Table 1:** Spray-dried proliposome formulations (F1 –F10) prepared using five different types of lactose carriers (Lactose monohydrate (LMH), lactose microfine (LMF), lactose 003, lactose 220 and lactose 300), two different solvents (water: Ethanol 50:50% v/v, and ethanol alone 100%) using spray drying parameters, n = 3.

Formulations	Lactose Carrier	Solvent Water:Ethanol (v/v)	Inlet Temperature (°C)	Airflow Rate (L/h)	Pump Rate (%)	Aspirator (%)
F1	LMH	1:1	120	601	15	100
F2	LMF	1:1	120	601	15	100
F3	Lactose 003	1:1	120	601	15	100
F4	Lactose 220	1:1	120	601	15	100
F5	Lactose 300	1:1	120	601	15	100
F6	LMH	0:1	120	601	15	100
F7	LMF	0:1	120	601	15	100
F8	Lactose 003	0:1	120	601	15	100
F9	Lactose 220	0:1	120	601	15	100
F10	Lactose 300	0:1	120	601	15	100

For formulations F1-F5 each lactose carrier (3000 mg) was dissolved in aqueous solvent (50 ml of distilled water) and the lipid phase (containing SPC (500 mg) and BDP (50 mg)) into organic solvent (50 ml of ethanol). Both solutions (aqueous and organic solutions) were combined in 150 ml beaker using a magnetic stirrer (Benchmark Scientific, UK) at 300 RPM for 5 min.

However, for SDP formulations F6-F10 the aqueous medium was omitted. Instead, the carbohydrate carrier along with SPC and BDP were all dispersed in organic solvent (ethanol 100%), followed by spray drying employing similar parameters (Table 1).

Upon preparation, each formulation was then spray-dried using a 0.7 mm diameter spray dryer nozzle, employing 100% aspirator to manufacture SDP formulations (Table 1).

Post-spray drying, SDP powders were collected in a collecting chamber and the production yield was determined with the help of below equation.


$$Production\;Yield\;\left(\%\right)=\left(\frac{Wf-We}{Wi}\right)\;x\;100$$

Where, “*Wf”* is the final mass of SDP powder collected after spray drying, “*We”* is the mass of empty collecting chamber, and “*Wi”* is the initial mass (containing lactose carrier, SPC and BDP) before spray drying.

### Surface morphology using scanning electron microscopy

Surface morphology and particle size (~100 particles) of coarse lactose carriers and SDP powder formulations (F1-F10) were examined using scanning electron microscopy (SEM) (XL20 Inspect S, Philips). A sample of each formulation or coarse powder was placed onto a carbon coated aluminium stub, followed by two minutes of gold coating using a sputter coater (Emitech K550x Gold Sputter Coater). Upon observation, several images were taken of each sample were taken.

### Crystallinity studies using X-ray diffraction

Corse lactose carriers, BDP and SDP powder formulations (F1 –F10) were studied *via* X-ray diffraction (XRD) (Rigaku Miniflex, Rigaku Ltd., Japan) using a diffracted beam monochromator with Cu-Kα (λ = 0.154nm). Each sample was loaded onto a silicon standard sample holder and the intensity of diffraction was recorded at an angle of two theta between the angular ranges of 5–55° using a scan rate of 2°/min. The experiments were conducted using a power of 30 kV voltage and 15 mA current. The scan peaks from individual lactose carriers, BDP and powder formulations were examined and compared with respect to their amorphous and crystalline form.

### Moisture analysis using Thermogravimetric analysis

Thermogravimetric analysis (TGA Q50, TA Instruments, DE) was performed on each coarse lactose carrier and SDP powder formulations (F1 –F10) to determine moisture content. Individual samples (~5–10 mg) were placed onto a platinum pan followed by their loading into the furnace. The furnace temperature was gradually increased from 25°C to 180°C, at a constant rate of 20°C/min in a nitrogen environment to observe any change in the mass of sample loaded.

### Hydration and analysis of particle size, polydispersity index and zetapotential

SDP powder formulations (F1-F10) were hydrated in deionised water. In order to generate liposome suspensions, 240 mg of SDP powder was hydrated in 8 ml of deionized water, followed by 2 min of vortex mixing (Fisons WhirliMixer, Fisons Scientific Equipment, UK) to ensure complete dissolution of lactose carrier particles and dispersion/hydration of the lipid phase. Liposomes were than left for 2 hours annealing time at room temperature to stabilize liposome vesicles. Annealing time is theorised to overcome possible structural defects of liposome bilayers, post lipid phase hydration [46].

Post-annealing time, the volume median diameter (VMD; 50% undersize, also use interchangeably as an average particle size) and polydispersity index (PDI) were measured using Dynamic Light Scattering (DLS), *via* a Zetasizer Nanoseries instrument (Malvern Instruments Ltd., UK). Zeta potential of liposomes was also analyzed *via* Laser Doppler Velocimetry (LDV) employing Zetasizer Nanoseries, where the electrophoretic mobility of liposomes was determined.

### Entrapment efficiency of BDP

Each SDP powder formulation (240 mg) was hydrated with deionised water (8 ml) followed by 2 min of vortex mixing (Fisions WhirliMixer, Fisions Scientific Equipments, UK) in order to generate liposome suspensions. The resultant liposome suspension (0.5 ml) was pipetted into a Millipore centrifuge filter tube (10 KD) and subjected to bench centrifugation (Spectrafuge 24D, Labnet International, USA) was conducted for 30 minutes at 15,500g (13,000 RPM). This procedure allowed the separation of free or unentrapped drug through the Millipore filter, sedimenting at the bottom of the tube as filtrate. Whereas the entrapped drug in the liposome vesicles were extracted by the Millipore filter (liposome vesicles are too large to pass through the Millipore filter). The unentrapped BDP was analysed using high performance liquid chromatography (HPLC) (Agilent 1200 HPLC instrument, UK). The entrapment efficiency of BDP was determined *via* total drug, where 1 ml of liposome suspension was dissolved in 10 ml of methanol followed by HPLC quantification using the following equation.


$$Entrapment\;efficiency\;(\%)=\frac{Total\;drug-Unentrapped\;drug}{Total\;drug}x\;100$$

For quantification of BDP, a HPLC fitted with a UV detector was used for the analysis of sample at 239 nm (Agilent 1200 HPLC instrument, UK). Methanol and deionized water (75:25 v/v) were employed as a mobile phase, with a flow rate of 1.7 ml/min. An Agilent column C-18, 5 μm, 150 nm × 4.6 mm (Agilent Technology, UK) was used as a stationary phase. An injection volume of 20 μl was utilised with a column temperature of 40°C.

The drug recovery of SDP powder formulations was determined using the following equation.


$$Drug\;Recovery\;\left(\%\right)=\left(\frac{Pw}{Wi}\right)\;x\;100$$

Where, “*Wi*” is the initial mass (containing lactose carrier, SPC and BDP) before spray drying and “*Pw*” is the practical weight of the drug post spray drying (quantified by HPLC).

### Deposition of SDP powder in the next generation impactor using dry powder inhaler

A next generation impactor (NGI) (Copley Scientific Limited, UK) was employed using a dry powder inhaler (DPI) device (low resistant inhaler RS01; Plastiape, Italy) to investigate the performance and deposition profile of SDP powder formulations. Prior to operation, the NGI plates were washed (with water and then ethanol to eliminate contamination) and air dried in a fume hood. Each NGI plate was coated with tween 20 and ethanol (1% v/v) and left for 2 hours drying, to form a layer on the plate (to minimize particle bouncing on the plates during aerosolization). The NGI was attached with a Copley TPK 2000 critical flow controller (Copley Scientific, UK) and a Copley HCP5 vacuum pump (Copley Scientific, UK). A Copley DFM 2000 flow meter (Copley Scientific, UK) was used to adjust the air flow at 60 L/min. The effective aerodynamic cut-off diameter for each impaction stage at 60 L/min was calibrated by the manufacturer and stated as Stage 1 (8.06 μm); Stage 2 (4.46 μm); Stage 3 (2.82 μm); Stage 4 (1.66 μm); Stage 5 (0.94 μm); Stage 6 (0.55 μm); Stage 7 (0.34 μm) and Micro-orifice collector (MOC) (<0.34 μm).

Each SDP powder formulation (140 mg) was filled into seven hydroxypropyl methylcellulose (HPMC) (size 3; Qualicaps, Spain) hard capsules. Each filled capsule was loaded into DPI device which was then connected to the mouthpiece adaptor (i.e., induction port) of the NGI. For each SDP powder formulation, the NGI was adjusted with a flow rate of 60 L/min and delay time of 15 seconds by the flow controller before piercing the filled capsule in DPI device (*via* two pins). The capsule content was pulled by the negative pressure into the NGI plates for 5 seconds. Post-aerosolization of the SDP powder formulations, powder deposited in each stage was washed with methanol and water (75:25% v/v) to determine BDP concentration *via* HPLC. The same procedure was repeated for other SDP powder formulations. Moreover, emitted dose (ED), fine particle fraction (FPF), fine particle dose (FPD), and respirable fraction (RF) were also calculated using the following equations. FPD is the weight/mass of particles that are less than 5 μm in size, within the total emitted dose of formulation. This denotes to the formulation fraction which deposits in stages 2–7, demonstrating the cut-off sizes on these stages (4.46–0.34 μm) with an airflow rate of 60 L/min in the NGI. FPF is the fraction of particles that are less than 5 μm associated with the emitted mass of formulation. Whereas RF is the fraction of those particles with aerodynamic diameter less than 5 μm and was stated as the percentage of FPD (where formulation particles deposit between stages 2 to 7) to the deposition of formulation on all stages (i.e., 1 to 8). Additionally, the mass median aerodynamic diameter (MMAD) was also determined employing Copley Inhaler Testing Data Analysis Software (CITDAS) Version 3.10.


$$ED(\%)=\left(\frac{Initial\;mass\;in\;capsules-Final\;mass\;remaining\;in\;capsules}{Initial\;mass\;in\;capsules}\right)\;x\;100$$

FPD(mg)=MassofparticlesonStage2→Stage7

$$FPF\;(\%)=\left(\frac{FPD}{Initial\;mas\;sloaded\;into\;capsules}\right)\;x\;100$$

$$RF\;(\%)=\left(\frac{Mass\;of\;particles\;on\;Stage\;2\to 7}{Total\;particle\;mass\;on\;all\;stages}\right)\;x\;100$$

### Statistical analysis

All experiments were conducted in triplicate using three different SDP formulation batches. For statistical analysis one-way analysis of variance (ANOVA) or Student’s *t* test was used for comparing more than two groups or two sets of data, respectively. The value of *p* lower than 0.05 was deemed to be statistically significant.

## Results and discussion

### Surface morphology of SDP formulations

**Fig 1:**
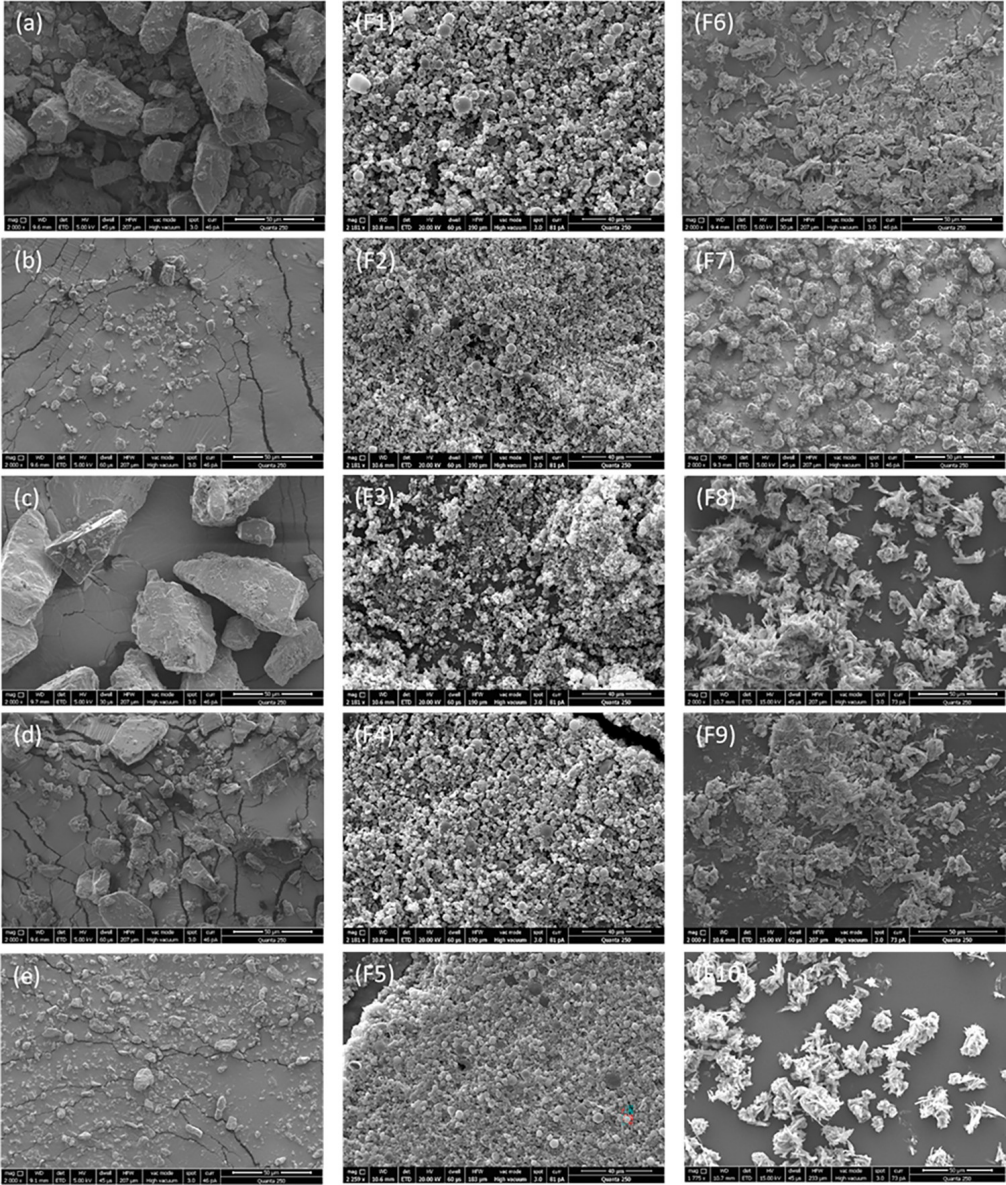
Scanning electron microscope images of coarse lactose carriers (Lactose monohydrate (LMH), lactose microfine (LMF), lactose 003, lactose 220 and lactose 300) (a-e), and spray-dried proliposome (SDP) powder formulations (F1-F5) using a dispersion medium of ethanol and water (50:50% v/v), and SDP powder formulations (F6-F10) when ethanol 100% was used as a dispersion medium. These images are typical of three such different experiments.

The surface morphology of various coarse lactose carriers was examined *via* scanning electron microscopy (SEM). Coarse lactose particles were observed to be non-porous and irregular in shape (Fig 1A–1E) [32]. However, when formulated as spray-dried proliposome (SDP) powders using a 50:50% v/v water to ethanol dispersion medium (Fig 1, F1-F5), particles were observed to be spherical in shape. Moreover, coarse lactose particles were larger in size (Fig 1A–1E) when compared to SDP formulations (F1-F5). Conversely, when only ethanol (100%) was employed as the dispersion medium for formulations F6-F10, SDP powders appeared to be irregular, agglomerated, oblong/ellipse and crystalline to needle in shape, as well as larger in size (Fig 1), making their potential deposition in the deep lung questionable. The difference in size and morphology observed between coarse lactose and formulations F1-F10, may thus be attributed to lactose carrier solubility in water, and lipid phase solubility in ethanol (Fig 1). These results are in agreement with previous research findings. For example, Thiyagarajan, Huck [47] demonstrated that when lactose carrier and their components were dissolved in their corresponding separate solvents, round particles were obtained. In terms of size, Omer, Hussein [48] observed a large particle size with irregular shape when lactose was employed as a carrier. Particle size and morphology may be attributed to the lower solubility of lactose carriers in ethanol, which may in turn result in crystallization/precipitation. As a consequence, SDP formulations F6-F10 were observed to be non-porous, whereas formulations F1-F5 exhibited porosity, which is deemed to be advantageous in terms of deep lung deposition in pulmonary drug delivery.

### Production yield of SDP formulations

**Fig 2:**
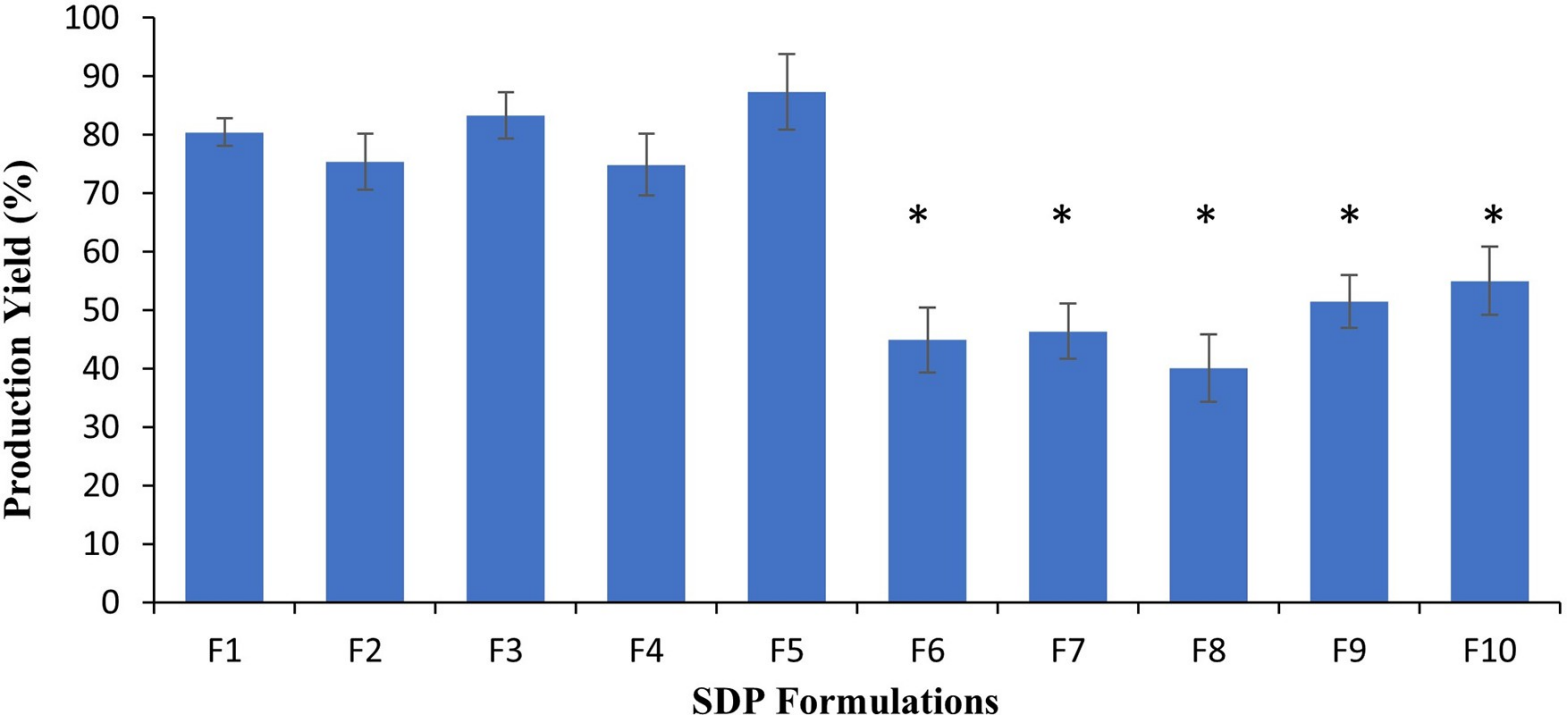
Production yield of spray-dried proliposome powder formulations when water and ethanol (50:50% v/v) was used as a dispersion medium (F1 –F5), and when ethanol alone (100%) was used as dispersion medium (F6 –F10). Data are mean ± STD, n = 3; *p<0.*05* for F6 –F10 compared to F1 –F5.

Upon analysis of the production yield, SDP powder formulations F1-F5 demonstrated significantly higher (*p<0*.*05*) production yield (75–87%) when compared to SDP powder formulations F6-F10 (40–55%) (Fig 2). Differences observed between the two groups of formulations (i.e., F1-F5 and F6-F10) may again be attributed to differences in dispersion medium, in the absence of any additional changes in parameters. In SDP formulations F1-F5, the carbohydrate carrier and lipid phase were separately and completely dissolved in their corresponding aqueous (i.e., water) and organic medium (i.e., ethanol), followed by their mixing to obtain a uniform dispersion; whereas in SDP F6-F10 formulations, the carbohydrate carrier was insoluble in organic solvent and therefore lactose particles remained suspended in ethanol, whilst the remainder of the lipid phase dissolved in the dispersion medium.

Upon aerosolization of formulations through the spray drying nozzle, uniform and consistent droplets were formed when F1-F5 formulations were used. This may also lead to a reduction in moisture content in these formulations (due to a high inlet temperature in drying chamber ~120°C) and an increase production yield [49, 50]. Whereas poor uniformity in droplets were formed due to the presence of undissolved lactose particles in dispersion medium for the F6-F10 formulations. It is noteworthy that greater uniformity in droplet size and morphology facilitates the formation of particles which are spherical and uniform in the drying chamber, in contrast to when precipitated lactose is present in the dispersion medium during spraying.

Differences in production yield (Fig 2) between the F1-F5 and F6-F10 formulations was confirmed *via* SEM (Fig 1). The differences observed may be attributed to particle density and morphology. With there being a propensity of higher density particles (F6-F10) to deposit on the internal walls of the spray-dryer chamber as opposed to porous, spherical particles which are likelier to reach the collection chamber, resulting in higher percentage yield (Fig 2).

### Particle size of SDP formulations

**Fig 3:**
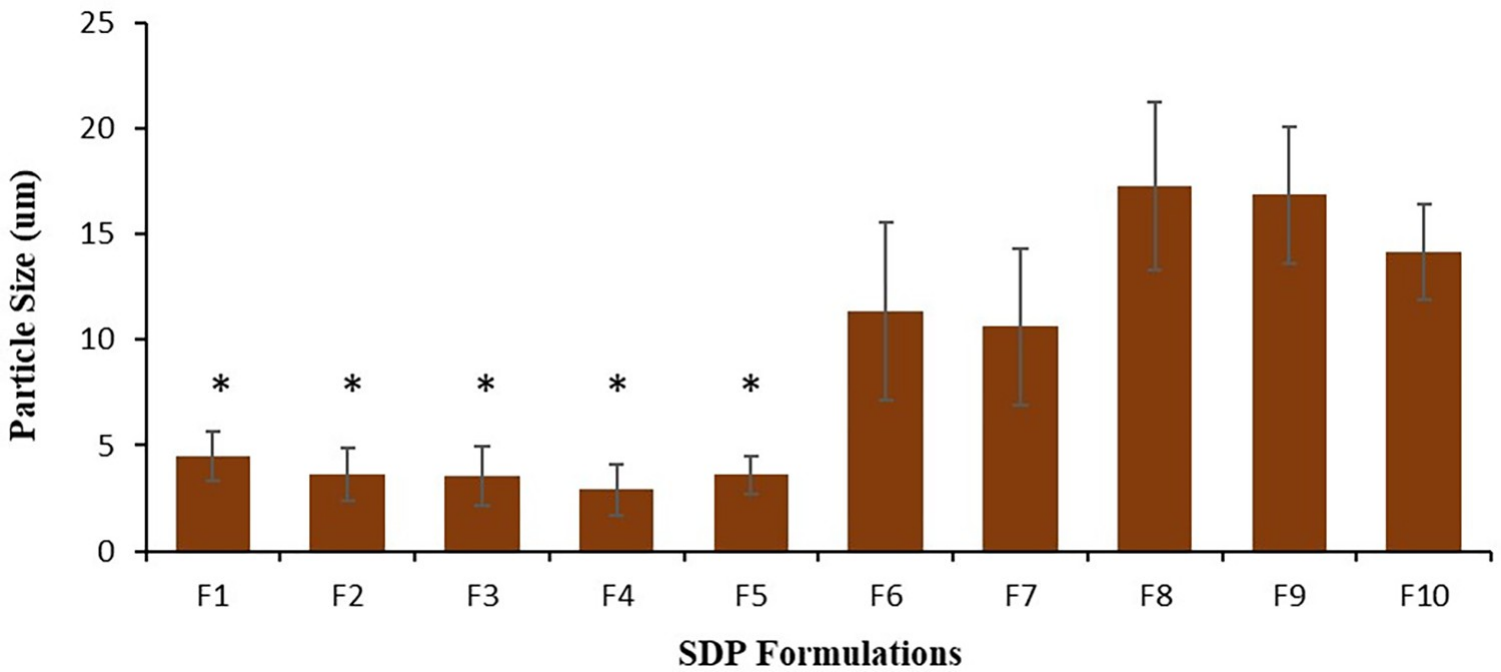
Particle size of spray-dried proliposome (SDP) powder formulations, where water and ethanol (50:50% v/v) was employed as a dispersion medium (F1 –F5), and SDP powder formulations (F6 –F10) when ethanol (100%) was used as dispersion medium. Data are mean ± STD, n = 3; *p<0.*05* for F1 –F5 compared to F6 –F10.

The measured particle size was determined upon SEM observation. SDP powders F1-F5 particle sizes were significantly lower (*P<0*.*05*) than the counterpart F6-F10 formulations (Fig 3). On average the particle size of SDP F1-F5 powders was 3.63 ± 0.19 μm, whereas for F6-F10 formulations this was determined to be 14.05 ± 0.78 μm (Fig 3). Similar to difference in morphology, differences in particle size are also associated with fluctuations observed production yield e.g., smaller particle size observed for the F1-F5 formulations was associated with a higher production yield than the counterpart F6-F10 formulations (Figs 2 and 3). Differences in dispersion media between the two groups of formulation are again deemed to be responsible for the differences observed.

### X-ray diffraction studies of SDP powder

**Fig 4:**
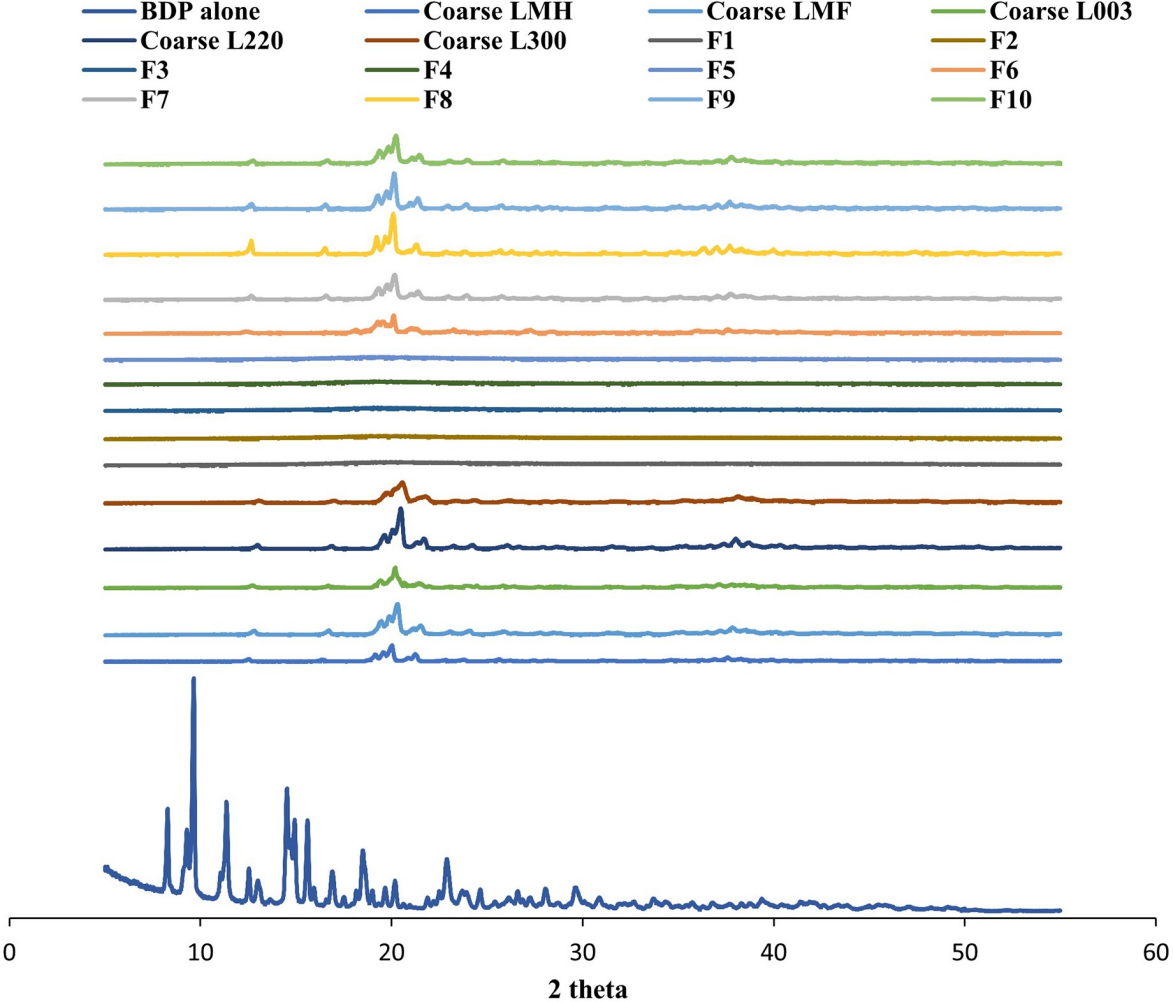
X-ray diffraction spectrum for BDP (alone), and five types of lactose carriers (Lactose monohydrate (LMH), lactose microfine (LMF), lactose 003, lactose 220 and lactose 300), followed by their incorporation and formulations of spray-dried proliposome (SDP) powder formulations (F1-F5) when water and ethanol (50:50% v/v) was used as a dispersion medium, and SDP powder formulations (F6-F10) when ethanol alone was used 100% as a dispersion medium. This data is typical of three such different experiments.

In order to determine the degree of crystallinity of the formulations developed, X-ray diffraction was employed. Initially, BDP was tested to identify its nature of state, which was noted to be crystalline (Fig 4). All five coarse lactose carriers were also confirmed as crystalline in structure, based on high intensity peaks observed for each (Fig 4). No visible peaks were observed for SDP formulations F1-F5, indicating amorphous nature of the powders. Contrastingly, high intensity peaks were observed for SDP formulations F6-F10, indicating crystallinity of the powders. Additionally, in all formulations tested, no peak was observable for the BDP incorporated, suggesting that this was present in the formulations in an amorphous form.

As the defining difference between the two formulations was dispersion media, again the differences observed may be attributed to this, irrespective of carrier type. Previous research also documents an increase in dry powder crystallinity when ethanol has been employed as a solvent [51–53] mirroring findings observed for SDP formulations F6-F10. Additionally, alternative research has also demonstrated that post-spray drying lactose particles are typically amorphous, when water is employed as the dispersion medium and conversely crystalline, when ethanol was employed [48].

### Moisture content of SDP formulations

**Fig 5:**
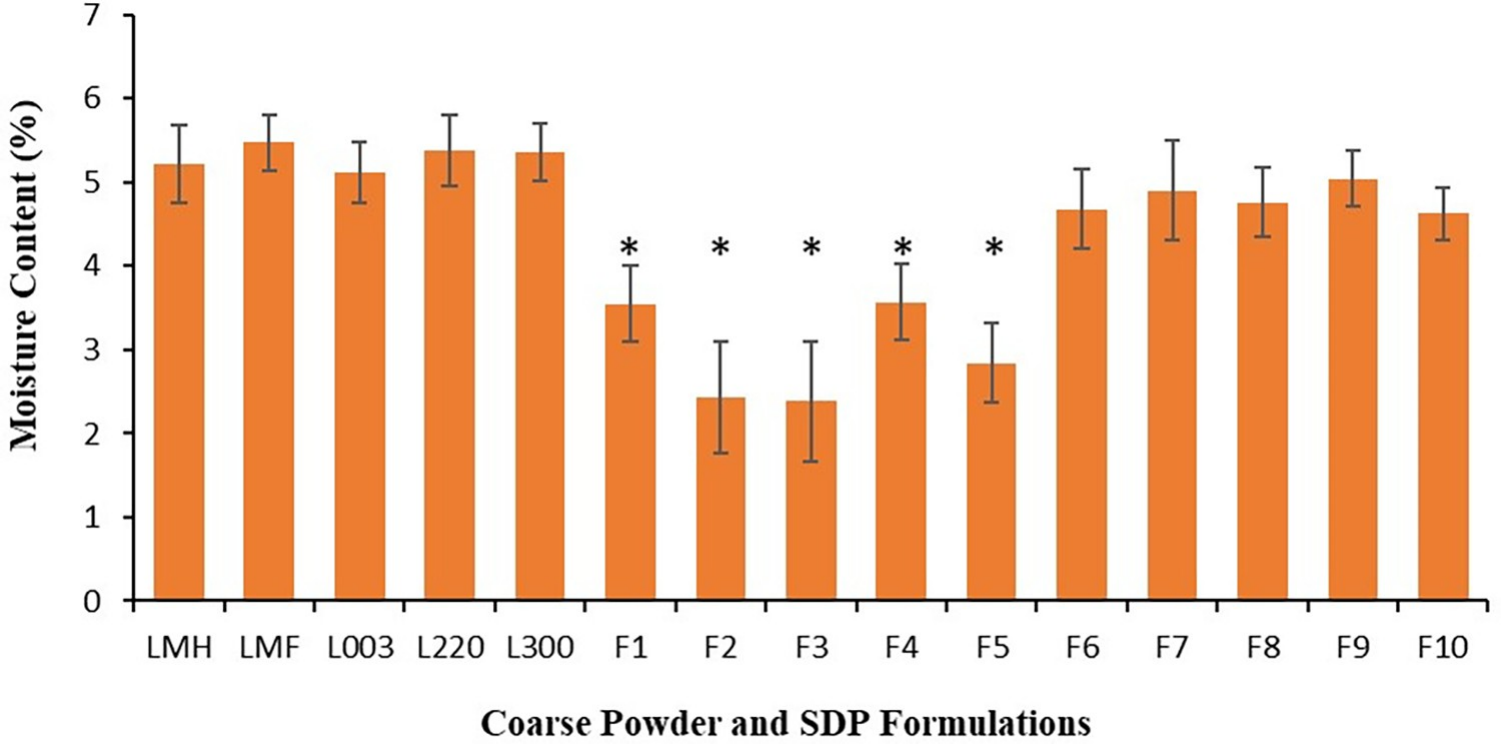
Moisture content of coarse lactose carriers (Lactose monohydrate (LMH), lactose microfine (LMF), lactose 003, lactose 220 and lactose 300), spray-dried proliposome (SDP) powders (F1-F5) where water and ethanol (50:50% v/v) was used as a dispersion medium, and SDP powders (F6-F10) where ethanol (100%) was only used as a dispersion medium. Data are mean ± STD, n = 3; *p<0.*05* for F1 –F5 compared to F6 –F10 and LMH, LMF, L003, L220, L300; *p>0*.*05* for F6 –F10 compared to LMH, LMF, L003, L220, L300.

The moisture content values for the SDP powder F1-F5 formulations were in agreement with literature values (2.43 ± 0.67–3.57 ± 0.46%) for particles which are purposed for lung deposition [54, 55]. This is in contrast to the F6-F10 formulations which elicited an elevated moisture content 4.62 ± 0.32–5.04 ± 0.33% (Fig 5), these values were more in line with the moisture content of coarse lactose carries i.e., 5.21 ± 0.46–5.37 ± 0.42% (Fig 5).

The significantly lower moisture content (*p<0*.*05*) observed for the SDP formulations F1-F5 may be attributed porosity of the formed particles and amorphous morphology, as a result of the high inlet temperature used (Fig 5). In contrast, coarse lactose carriers were noted to possess high moisture content, attributed to their crystalline structure (Fig 5). However, out of the formulations and carriers tests, the highest observed moisture content was for the of the SDP F6-F10 formulations, this was deemed to be due their poor dissolution in the ethanol medium, retaining crystallinity as confirmed by SEM and XRD (Figs 1 and 4). The higher residual moisture observed ay be contributory in reducing powder recovering as observed in Fig 2 and noted in previous research [50, 56].

### Entrapment efficiency of BDP, VMD, PDI and zeta potential of liposomes

Upon generation of liposomes *via* hydration of SDP powder formulations, volume median diameter (size) was analysed. Formulations F1-F5 produced significantly smaller (*p<0*.*05*) particles ranging from 126.65 ± 6.78 to 252.16 ± 8.46 μm, when compared to the counterpart F6-F10 formulations (Table 2), irrespective of lactose carrier type. SDP formulations prepared with ethanol alone (F6-F10) produced particles two-fold larger than F1-F5 formulations. Formulation PDI was also reflective of this difference, i.e., lower for F1-F5 formulations (<0.43 ± 0.06), and higher for F6-F10 (0.68 ± 0.05–0.92 ± 0.07) (*p<0*.*05*) (Table 2). Higher VMD is typically associated with higher PDI, this may be a result of vesicle fusion/aggregation which may cause interdigitation and hence produce particles with various ranges and possibly reduce entrapment efficiency [23, 57]. Both size and PDI are deemed to be influential factors in the retention time of encapsulated drug and sustained drug release [58].

**Table 2:** Generation of liposome from spray-dried proliposome (SDP) powder upon hydration in aqueous media were characterized via volume median diameter (VMD), polydispersity index (PDI), zeta potential, entrapment efficiency and drug recovery. SDP powder F1-F5 were prepared using water and ethanol (50:50% v/v ratio) as a dispersion medium, and SDP powder F6-F10 were prepared using ethanol (100%) as a dispersion medium. Data are mean ± STD, n = 3.

Formulations	VMD (μm)	PDI	Zeta potential (mV)	Entrapment efficiency (%)	Drug recovery (%)
**F1**	126.65 ± 6.78	0.37 ± 0.06	4.68 ± 1.77	95.45 ± 7.54	87.54 ± 6.72
**F2**	169.65 ± 7.55	0.36 ± 0.05	7.88 ± 1.82	96.89 ± 7.64	84.62 ± 5.28
**F3**	154.33 ± 8.45	0.29 ± 0.08	7.23 ± 1.97	94.67 ± 8.41	85.44 ± 7.16
**F4**	219.52 ± 7.74	0.42 ± 0.07	6.91 ± 1.33	96.35 ± 7.93	83.63 ± 6.59
**F5**	252.16 ± 8.46	0.43 ± 0.06	6.91 ± 1.75	95.26 ± 8.09	84.51 ± 5.08
**F6**	912.65 ± 9.41	0.68 ± 0.05	5.73 ± 1.69	82.95 ± 9.62	74.45 ± 4.17
**F7**	883.32 ± 10.58	0.87 ± 0.07	6.65 ± 1.64	79.21 ± 8.76	69.46 ± 4.58
**F8**	827.85 ± 10.26	0.72 ± 0.06	5.42 ± 1.47	80.21 ± 8.61	73.62 ± 5.24
**F9**	953.36 ± 9.08	0.71 ± 0.08	7.54 ± 1.79	78.16 ± 9.35	72.38 ± 5.23
**F10**	745.57 ± 10.11	0.92 ± 0.07	4.69 ± 1.81	80.17 ± 7.46	70.08 ± 4.78

Upon analysis of zeta potential values, no significant difference (*p>0*.*05*) was noted between any of the formulations (Table 2). One of the main components used which self-assemble and generate vesicles is a phospholipid (i.e., SPC). SPC is a zwitterion compound with an isoelectric point of 6–7, meaning that it is neutral in charge. Moreover, formulations prepared with SPC demonstrated slightly positive charge (Table 2). The presence of a charge could be beneficial in order to keep the formulations stable, as the electrostatic repulsive forces tend to reduce vesicle aggregation and fusion. However, the presence of slight charges is not substantially high and thus maybe be attributed to the trace amounts of impurity (the purity of phospholipids SPC is 94%; thus, the percentage impurity of phospholipids may cause a mild surface charge). Furthermore, the presence of a cationic compounds in the lipid-based vesicle may result in their positive charge and therefore improve their interaction with cell membranes containing negative charges [59, 60]. Additionally, cationic liposomes are considered a new generation of liposomes that increase circulation time, enhancing pulmonary absorption when compared to conventional liposomes [61].

The entrapment efficiency was marginally higher for F1-F5 formulations ranging from 94.67 ± 8.41–96.35 ± 7.93%, when compared to F6-F10 formulations (78.16 ± 9.35–82.95 ± 9.62%) (Table 2), though this difference was not statistically significant. Table 2 also demonstrated the recovery values of BDP. The recovery of drug is higher in F1-F5 formulation when compared to F6-F10 formulations. This may suggest that the value of higher percentage recovery is dependent upon on the dispersion medium and irrespective of the carrier type used. Moreover, higher drug recovery is in parallel to higher production yield (Fig 2), which may be attributed to the higher ratio of carrier used to lipid phase [48]. The lower entrapment efficiency may be attributed to the lower concentration of phospholipid around the crystalline lactose carriers and higher concentration potentially deposited in the drying chamber of spray drying instrument [23, 48]. Drug loading is an important factor in formulation optimisation, which may be affected by the lipid to drug ratio. The higher the lipid ratio when compared to drug, the higher the drug loading [62]. This may be related to the greater space offered by vesicles to accommodate drug within lipid bilayers, and therefore reduce loss of drug (insufficient loading) [6]. Furthermore, the entrapment of drug using elevated temperatures may also improve drug loading, however this is also restricted by the temperature sensitivity of formulation excipients used [62]. The method of preparation (e.g., dehydration-rehydration) may also improve drug loading. However, it is important to know that drug concentration used in this study was not varied (i.e., 50 mg was employed in every formulation) to assess impact upon drug loading. Moreover, it is recognised that a number of strategies can be employed to enhance drug loading and vesicle uniformity, including the incorporation of an amphiphilic co-polymer which has been previously demonstrated by Lv, Wu [63] to improve both drug loading and uniformity when used in combination with drugs which possess electron-donating groups, this may be potentially be extended to BDP which is known to have a number of electron-donating groups.

The crystalline particles of SPD F6-F10 formulations (Fig 1) were noted to possess a smaller surface area when compared to the amorphous particles of F1-F5 formulations. Smaller, highly porous particles (i.e., formulations F1-F5) may contain higher concentrations of phospholipids. Formulations deemed as having higher concentrations of phospholipid (i.e., F1-F5) were noted to generate greater numbers of liposome vesicles. The aforementioned data indicates a correlation between particle morphology, production yield and drug entrapment efficiency, with F1-F5 formulation observed to be superior across these measures when compared to F6-F10 formulations.

### Aersolization performance of SDP powder formulations

SDP powders with spherical particles potentially may have superior flowability due to their morphology, this is likelier when combined with a low particle size (i.e., in respirable range). Such powders are more likely to deposit in the peripheral airways. Whilst SEM was employed in this study to observe size and morphology, it is not an indicator of how particles may behave aerodynamically. This is key, as deposition of particles in the pulmonary system is influenced by the particle aerodynamic size and shape [64], for example, particles smaller than 5 μm are more likely to reach the lower airways following inhalation [65, 66].

The *in-vitro* deposition properties of dry powders were determined using a next generation impactor (NGI). The ED (emitted dose) was high for all SDP powder formulations ranging from 78.55–93.64% (Table 3), irrespective of carrier type and dispersion media used. FPF represent those particles where the particle size is less than 5 μm, and they possess a high probability to deposit/penetrate the deep lung. Whereas, particles larger than this size may deposit into the oropharyngeal region, resulting in a lower FPF [67]. FPF demonstrated that the SDP powder F1-F5 formulations produced significantly higher FPF% (*p<0*.*05*) when compared to the F6-F10 formulations (Table 3). Larger particle size in combination with irregular shape and high density may further reduce FPF percentage deposition. SDP powder formulations F6-F10 demonstrated larger particle size, also observed and confirmed *via* SEM (Fig 1), resulting in lower particle deposition in the lateral stages of NGI. The highest FPF value was observed for the F5 formulation (33.42%), though when compared to formulations F1-F4 this difference was not statistically significant. This suggests that the performance of SDP powder is principally dependent on dispersion media, as opposed to other parameters varied e.g., carrier type. Moreover, the FPF values of F1-F5 formulations were seven times higher than F6-F10 (Table 3). These findings are in agreement with SEM observations, as all SDP powder formulations F1-F5, showed amorphous particles of small size, and non-porous crystalline particles for F6-F10 powders (Fig 1).

**Table 3:** Powder deposition performance including emitted dose (ED), fine particle dose (FPD), fine particle fraction (FPF), respirable fraction (RF) and mass median aerodynamic diameter (MMAD) of spray-dried proliposome (SDP) powder F1-F5 (prepared using a dispersion medium of water:ethanol in a 50:50% v/v ratio) and SDP of F6-F10 (when ethanol 100% was used as a dispersion medium) via next generation impactor (NGI) at 15 L/min airflow rate. Data are mean ± STD, n = 3.

Formulations	ED (%)	FPD (μg)	FPF (%)	RF (%)	MMAD (μm)
**F1**	93.64 ± 7.53	773.34 ± 152.71	28.45 ± 4.55	82.43 ± 10.57	3.49 ± 0.26
**F2**	88.54 ± 6.85	1054.99 ± 148.66	30.54 ± 6.09	89.47 ± 10.23	3.19 ± 0.25
**F3**	90.34 ± 10.54	765.89 ± 162.56	27.62 ± 7.12	81.77 ± 7.67	3.43 ± 0.21
**F4**	91.55 ± 9.07	888.89 ± 151.76	31.72 ± 4.92	89.14 ± 9.13	2.98 ± 0.28
**F5**	87.28 ± 11.35	968.13 ± 148.24	33.42 ± 5.53	86.71 ± 7.98	3.25 ± 0.23
**F6**	85.45 ± 8.82	99.01 ± 27.19	3.08 ± 0.84	19.12 ± 3.42	N/A
**F7**	90.25 ± 12.45	116.85 ± 28.43	5.02 ± 1.06	25.85 ± 5.04	N/A
**F8**	84.54 ± 10.22	77.53 ± 26.49	2.68 ± 0.64	21.46 ± 4.22	N/A
**F9**	82.47 ± 9.35	107.69 ± 24.98	4.39 ± 1.16	23.44 ± 6.71	N/A
**F10**	78.55 ± 8.45	100.62 ± 23.49	5.20 ± 1.42	28.25 ± 6.95	N/A

A similar pattern was observed for the FPD, where the FPD was significantly higher (*p<0*.*05*) for all F1-F5 SDP formulations when compared to F6-F10 (Table 3). On average, 42.47 mg of FPD was recorded for F1-F5 formulations and only 5.98 mg for F6-F10 formulations. This trend of higher deposition is attributed to particle morphology (amorphous, round and smaller particle size) of F1-F5 (Figs 1 and 2), which indicated that these particles are porous and lighter in weight, hence travelling further in the NGI stages. This is further reflected in the elevated (*p<0*.*05*) percentage of RF observed for the same F1-F5 SDP powder formulations (Table 3). Moreover, the crystalline and non-porous particles possess a greater density, acting as a barrier to deposition in the lower stages of the NGI, with associated low FPF and RF. Mass median aerodynamic diameter (MMAD) indicates that particles ≤ 5 μm in size are able to reach to the alveolar region for peripheral drug deposition. MMAD is the aerodynamic diameter where 50% of the aerosolised mass are below and above the stated diameter. All the SDP powder formulations tested (F1-F5), demonstrated a trend of smaller particle size for MMAD, with no significant difference (*p<0*.*05*) between formulations. However, no results were observed for the F6-F10 formulations, this may be related to the extremely low deposition of these formulation in various stages of the NGI. The values obtained for F1-F5 formulations indicated their high efficiency in depositing formulations at the alveolar region.

The FPF, FPD, RF and MMAD values obtained from SDP F1-F5 formulations were indicative of desirable aerosolization properties and a deep lung deposition profile. Hence, in theory, when inhaled, these proliposome powder formulations (F1-F5) will dissolve in the lung’s lining fluid producing liposomes, subsequently extending the release profile of BDP to be taken up by the lungs cells exhibiting biological response.

## Conclusions

In this study, SDP powder formulations (F1-F10) were designed and prepared using five different types of lactose carriers and two different dispersion media (water and ethanol in 50:50% v/v; and ethanol alone as 100%) for spray drying. Post-spray drying, SDP formulations F1-F5 (prepared with water and ethanol) produced significantly smaller and amorphous particles with comparatively higher entrapment values using BDP as the model drug, when compared to their counterpart F6-F10 formulations. This is indicative that the physicochemical properties of SDP powder formulations may be manipulated by altering dispersion media. Employing water and ethanol as dispersion medium (50:50% v/v ratio), resulted in significantly higher production yield, irrespective of lactose carrier. Following aerosolization performance using all F1-F10 SDP formulations, again formulations prepared with water and ethanol (F1-F5) as dispersion medium exhibited higher deposition, regardless of lactose carrier type. Moreover, F1-F5 were deposited with greater efficiency (based on higher FPF, FPD, RF and MMAD in the NGI stages). Thus overall, superior characteristics and performance were noted for F1-F5 formulations, which were prepared using water and ethanol as a dispersion medium.

## Supporting information

S1 Data (DOCX)
