# Supplementary material for: Impact of dispersion media and carrier type on spray-dried proliposome powder formulations loaded with beclomethasone dipropionate for their pulmonary drug delivery via a next generation impactor
Source: PLoS One. 2023 Mar 13;18(3):e0281860. doi: 10.1371/journal.pone.0281860 (PMC10010524; doi:10.1371/journal.pone.0281860)
Supplement: S1 Data — (DOCX) [file pone.0281860.s001.docx]

**Supplementary Data**

Upon analysis of the weight of the SDP powder formulations in stage 1 of NGI, formulations F1-F5 demonstrated significantly lower (p<0.05) powder deposition when compared to F6-F10 formulations (SFig 1). This may be attributed to the smaller particle size of SDP F1-F5, evading stage 1 due to the cut-off diameter (8.06 µm), depositing larger particles of F6-F10 formulations (also confirmed via SEM in Fig 3). However, upon reaching to stage 2 to 8 of NGI, where the cut-off diameter of NGI at 60 L/min airflow rate is 4.46-<0.34 µm; a significantly higher (p<0.05) amount of SDP powder was deposited for the F1-F5 when compared to the F6-F10 formulations. This may be related to the uniform and smaller particle size of these formulations with respect to irregular to larger particle size of F6-F10 formulations (Fig 3).

In terms of BDP concentration in each stage of the NGI, similar results were found where a higher mass of PDP powder demonstrated higher concentration of BDP, and a lower amount of SDP powder represented lower BDP concentration (SFig 1).

The cumulative percentage of BDP was determined from the SDP formulations F1-F10, where stage 1 of NGI showed significantly higher (p<0.05) percentage of BDP for F6-F10 formulations when compared to the counterpart F1-F5 formulations (SFig 1). This showed higher powder deposition in stage 1 containing higher BDP (where particle size was more than 8.06 µm), whereas F1-F5 formulations only deposited a small mass of SDP powder due to the smaller particle size and therefore attributed to lower BDP percentage deposited in stage 1. The cumulative percentage of BDP showed a higher increase in formulations F6-F10 than compared to F1-F5 (SFig 1).


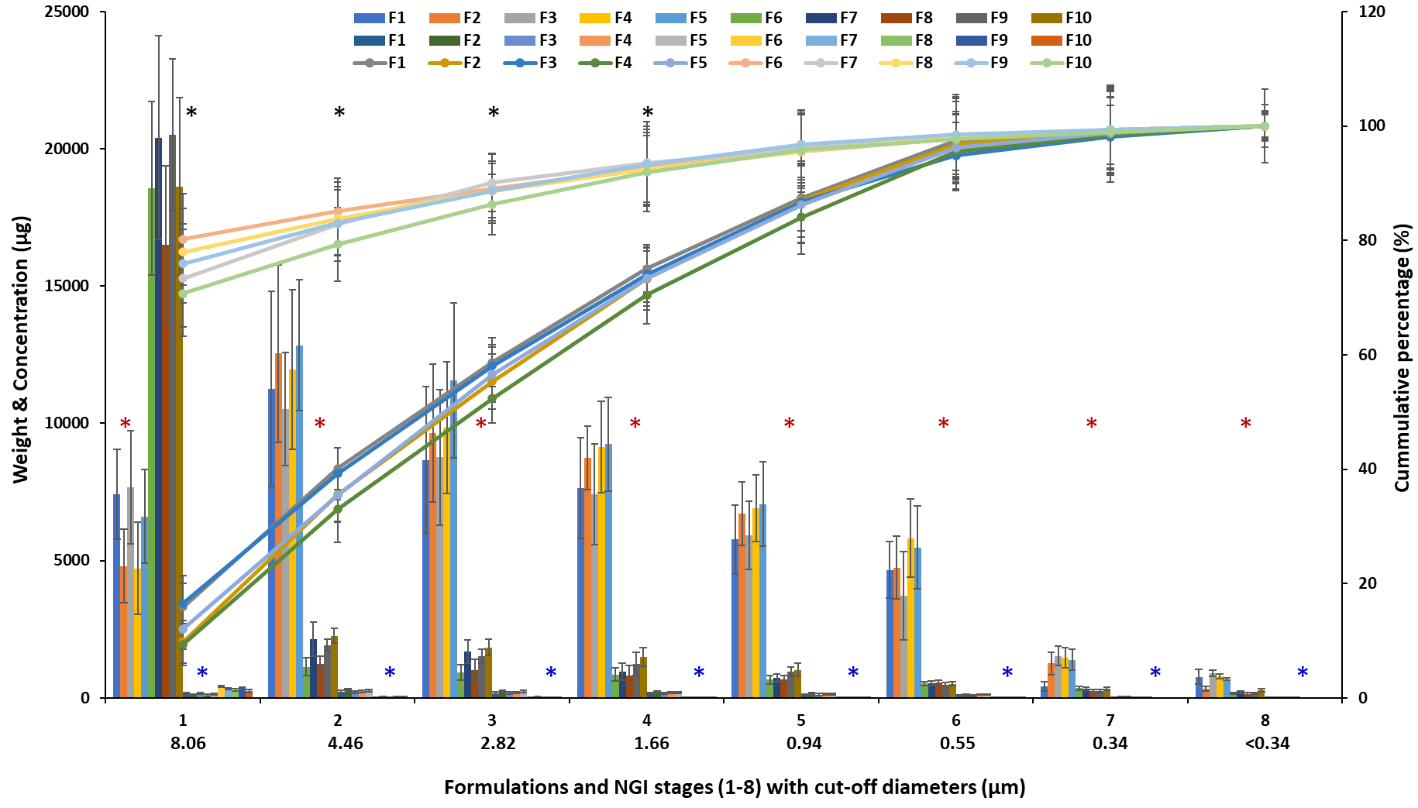


**SFig 1. Weight of powder and concentration of BDP (vertical bars), and cumulative percentage of BDP concentration (horizontal lines) of spray-dried proliposome formulations (F1-F10) recovered post aerosolization using next generation impactor, containing stages 1-8 along with their cut-off diameter (8.06-<0.34 µm) at an airflow rate of 60L/min. Where water and ethanol (50:50% v/v) was used as a dispersion medium (F1-F5), and when ethanol alone (100%) was used as dispersion medium (F6-F10). Data are mean ± STD, n = 3. In weight** **(dark red steric), *p<0.05 for F1-F5 compared to F6-F10 at stage 1, whereas *p<0.05 for F6-F10 compared to F1-F5 for stages 2-8. In concentration** **(dark blue steric), *p<0.05 for F1-F5 compared to F6-F10 at stage 1, whereas *p<0.05 for F6-F10 compared to F1-F5 for stages 2-8. In cumulative percentage of BDP concentration (black steric), *p<0.05 for F1-F5 compared to F6-F10 at stage 1-4, whereas *p>0.05 for F1-F5 compared to F6-F10 at stage 5-8**
